# Supplementary figures and images for: Characterization of the chloroplast genome of Lonicera ruprechtiana Regel and comparison with other selected species of Caprifoliaceae
Source: PLoS One. 2022 Jan 25;17(1):e0262813. doi: 10.1371/journal.pone.0262813 (PMC8789150; doi:10.1371/journal.pone.0262813)

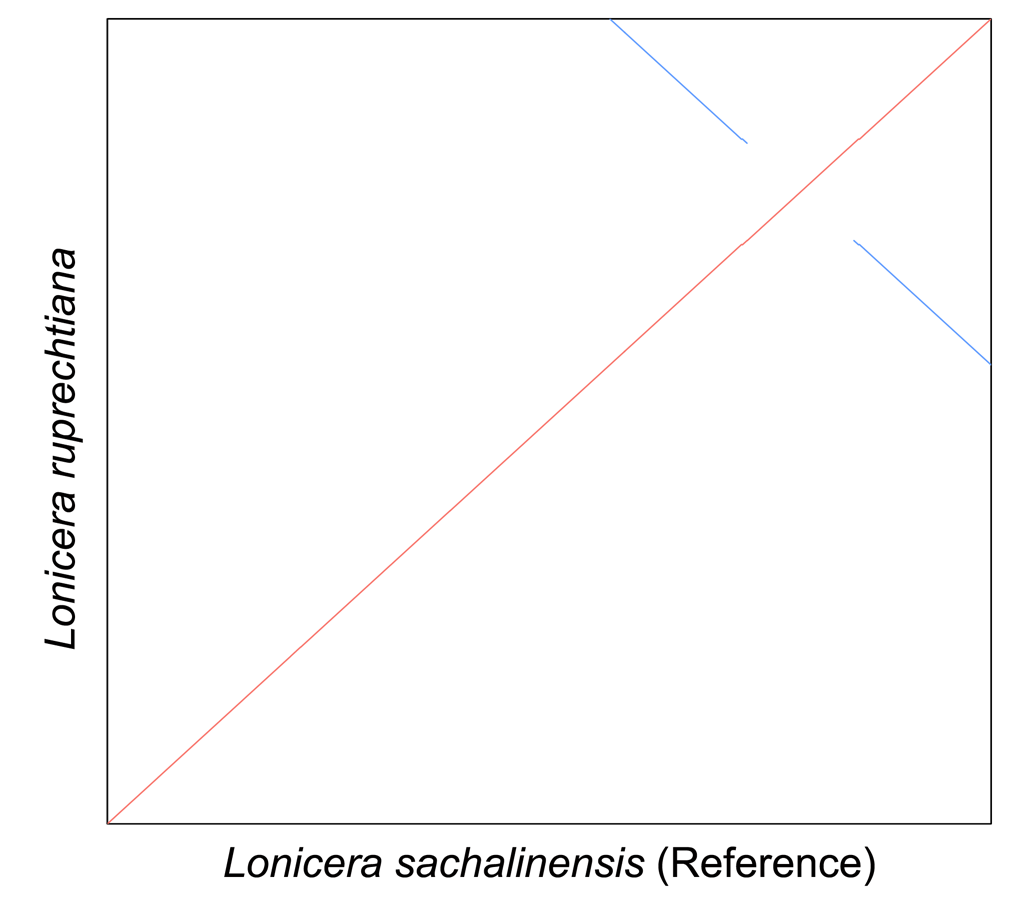

Supplement: S1 Fig — Red is the result in the same direction; blue is the result in the opposite direction. (TIF) [file pone.0262813.s001.tif]
