# Supplementary material for: Characterization of the chloroplast genome of Lonicera ruprechtiana Regel and comparison with other selected species of Caprifoliaceae
Source: PLoS One. 2022 Jan 25;17(1):e0262813. doi: 10.1371/journal.pone.0262813 (PMC8789150; doi:10.1371/journal.pone.0262813)
Supplement: S3 Table — (DOCX) [file pone.0262813.s004.docx]

**Table S3. Repeat sequences of *L. ruprechtiana* cp genome**

| **Number** | **Repeats size** | **First repeat start** | **location** | **Repeat types** | **Second repeat start** | **location** |
| --- | --- | --- | --- | --- | --- | --- |
| 1 | 287 | 111753 | no gene | Palindromic | 130753 | no gene |
| 2 | 75 | 38268 | no gene | Palindromic | 110566 | *trnG-GCC* |
| 3 | 75 | 38268 | no gene | Forward | 132152 | no gene |
| 4 | 71 | 38272 | no gene | Palindromic | 110566 | *trnG-GCC* |
| 5 | 71 | 38272 | no gene | Forward | 132156 | no gene |
| 6 | 60 | 111117 | no gene | Forward | 111212 | no gene |
| 7 | 60 | 111117 | no gene | Palindromic | 131521 | no gene |
| 8 | 60 | 111212 | no gene | Palindromic | 131616 | no gene |
| 9 | 60 | 131521 | no gene | Forward | 131616 | no gene |
| 10 | 82 | 71015 | no gene | Forward | 71036 | *rps18* |
| 11 | 70 | 131511 | no gene | Forward | 131606 | no gene |
| 12 | 66 | 300 | no gene | Palindromic | 300 | no gene |
| 13 | 79 | 60077 | *accD* | Forward | 60104 | *accD* |
| 14 | 71 | 60085 | *accD* | Forward | 60112 | *accD* |
| 15 | 82 | 71023 | *rps18* | Forward | 71044 | *rps18* |
| 16 | 83 | 128732 | *ycf1* | Forward | 128855 | *ycf1* |
| 17 | 99 | 128716 | *ycf1* | Forward | 128839 | *ycf1* |
| 18 | 90 | 128725 | *ycf1* | Forward | 128848 | *ycf1* |
| 19 | 72 | 128743 | *ycf1* | Forward | 128866 | *ycf1* |
| 20 | 117 | 151973 | *ycf2* | Forward | 151997 | *ycf2* |
| 21 | 120 | 151970 | *ycf2* | Forward | 151994 | *ycf2* |
| 22 | 110 | 90679 | *ycf2* | Forward | 90703 | *ycf2* |
| 23 | 110 | 90679 | *ycf2* | Palindromic | 151980 | *ycf2* |
| 24 | 110 | 90703 | *ycf2* | Palindromic | 152004 | *ycf2* |
| 25 | 110 | 151980 | *ycf2* | Forward | 152004 | *ycf2* |
| 26 | 113 | 151977 | *ycf2* | Forward | 152001 | *ycf2* |
| 27 | 96 | 151970 | *ycf2* | Forward | 152018 | *ycf2* |
| 28 | 93 | 90679 | *ycf2* | Forward | 90727 | *ycf2* |
| 29 | 93 | 90679 | *ycf2* | Palindromic | 151973 | *ycf2* |
| 30 | 93 | 90727 | *ycf2* | Palindromic | 152021 | *ycf2* |
| 31 | 93 | 151973 | *ycf2* | Forward | 152021 | *ycf2* |
| 32 | 89 | 151977 | *ycf2* | Forward | 152025 | *ycf2* |
| 33 | 86 | 151980 | *ycf2* | Forward | 152028 | *ycf2* |
| 34 | 67 | 149979 | *ycf2* | Forward | 150015 | *ycf2* |
| 35 | 64 | 92711 | *ycf2* | Forward | 92747 | *ycf2* |
| 36 | 64 | 92711 | *ycf2* | Palindromic | 149982 | *ycf2* |
| 37 | 64 | 92747 | *ycf2* | Palindromic | 150018 | *ycf2* |
| 38 | 64 | 149982 | *ycf2* | Forward | 150018 | *ycf2* |
| 39 | 71 | 149975 | *ycf2* | Forward | 150011 | *ycf2* |
| 40 | 59 | 90730 | *ycf2* | Forward | 90754 | *ycf2* |
| 41 | 59 | 90730 | *ycf2* | Palindromic | 151980 | *ycf2* |
| 42 | 59 | 90754 | *ycf2* | Palindromic | 152004 | *ycf2* |
| 43 | 70 | 92705 | *ycf2* | Forward | 92741 | *ycf2* |
| 44 | 70 | 92705 | *ycf2* | Palindromic | 149982 | *ycf2* |
| 45 | 70 | 92741 | *ycf2* | Palindromic | 150018 | *ycf2* |
| 46 | 55 | 90734 | *ycf2* | Forward | 90758 | *ycf2* |
| 47 | 55 | 90734 | *ycf2* | Palindromic | 151980 | *ycf2* |
| 48  49 | 55  58 | 90758  152032 | *ycf2*  *ycf2* | Palindromic  Forward | 152004  152056 | *ycf2*  *ycf2* |
