# Supplementary material for: Characterization of the chloroplast genome of Lonicera ruprechtiana Regel and comparison with other selected species of Caprifoliaceae
Source: PLoS One. 2022 Jan 25;17(1):e0262813. doi: 10.1371/journal.pone.0262813 (PMC8789150; doi:10.1371/journal.pone.0262813)
Supplement: Attachment — Submitted filename: Response to reviewers.docx [file pone.0262813.s007.docx]

December 24, 2021

Dr Branislav T. Šiler
Academic Editor 
PLOS ONE

Dear Editor and Reviewers,

RE: Response to Decision Letter for Manuscript ID. PONE-D-21-23416

First, thank you for your comments. We have now revised our manuscript further in accordance with your recommendations.

The following is the response to the comments of the academic editor and the reviewers with a detailed and clear point-by-point response.

We are looking forward to your positive decision.

Sincerely yours,

Qingbei Weng,

Ph.D, Professor

Department of Biotechnology

School of Life Sciences,

Guizhou Normal University, Guiyang, China

Manuscript ID. PONE-D-21-23416

Dear Dr. Weng,

Thank you for submitting your manuscript to PLOS ONE. After careful consideration, we feel that it has merit but does not fully meet PLOS ONE’s publication criteria as it currently stands. Therefore, we invite you to submit a revised version of the manuscript that addresses the points raised during the review process.

==============================

From the Reviewers' reports, it is obviuos that numerous methodological solutions were not properly chosen or at least were not satisfactorily introduced or explained. Please provide a detailed Response to Reviewers stating each of their concerns along with the intervention made in the revised version of the manuscript. Also, be aware of additional remarks provided in the attachment by Reviewer #1.

==============================

Please submit your revised manuscript by Oct 30 2021 11:59PM. If you will need more time than this to complete your revisions, please reply to this message or contact the journal office at plosone@plos.org. When you're ready to submit your revision, log on to https://www.editorialmanager.com/pone/ and select the 'Submissions Needing Revision' folder to locate your manuscript file.

Please include the following items when submitting your revised manuscript:

A rebuttal letter that responds to each point raised by the academic editor and reviewer(s). You should upload this letter as a separate file labeled 'Response to Reviewers'.

A marked-up copy of your manuscript that highlights changes made to the original version. You should upload this as a separate file labeled 'Revised Manuscript with Track Changes'.

An unmarked version of your revised paper without tracked changes. You should upload this as a separate file labeled 'Manuscript'.

If you would like to make changes to your financial disclosure, please include your updated statement in your cover letter. Guidelines for resubmitting your figure files are available below the reviewer comments at the end of this letter.

If applicable, we recommend that you deposit your laboratory protocols in protocols.io to enhance the reproducibility of your results. Protocols.io assigns your protocol its own identifier (DOI) so that it can be cited independently in the future. For instructions see: https://journals.plos.org/plosone/s/submission-guidelines#loc-laboratory-protocols. Additionally, PLOS ONE offers an option for publishing peer-reviewed Lab Protocol articles, which describe protocols hosted on protocols.io. Read more information on sharing protocols at https://plos.org/protocols?utm_medium=editorial-email&utm_source=authorletters&utm_campaign=protocols.

We look forward to receiving your revised manuscript.

Kind regards,

Branislav T. Šiler, Ph.D.

Academic Editor

PLOS ONE

Journal requirements:

When submitting your revision, we need you to address these additional requirements.

1. Please ensure that your manuscript meets PLOS ONE's style requirements, including those for file naming. The PLOS ONE style templates can be found at

https://journals.plos.org/plosone/s/file?id=wjVg/PLOSOne_formatting_sample_main_body.pdf and

https://journals.plos.org/plosone/s/file?id=ba62/PLOSOne_formatting_sample_title_authors_affiliations.pdf

Response: Thank you for your comment. We have changed our manuscript to meet PLOS ONE's style requirements.

2. Thank you for stating the following financial disclosure:

“This study was supported by Provincial Program on Platform and Talent Development of the Department of Science and Technology of Guizhou China under Grant [No. [2019]5655 and [2019]5617], and the Guizhou Provincial Science and Technology Foundation under Grant (No. 2020–1Y096).”

Please state what role the funders took in the study. If the funders had no role, please state: "The funders had no role in study design, data collection and analysis, decision to publish, or preparation of the manuscript."

If this statement is not correct you must amend it as needed.

Please include this amended Role of Funder statement in your cover letter; we will change the online submission form on your behalf.

Response: Thank you for your comment. We have added the statement the role of funders took in the study and author contributions in cover letter.

3. Thank you for stating the following in the Funding Section of your manuscript:

“This study was supported by Provincial Program on Platform and Talent Development of the Department of Science and Technology of Guizhou China under Grant [No. [2019]5655 and [2019]5617], and the Guizhou Provincial Science and Technology Foundation under Grant (No. 2020–1Y096).”

Please note that funding information should not appear in other areas of your manuscript. We will only publish funding information present in the Funding Statement section of the online submission form.

Please remove any funding-related text from the manuscript and let us know how you would like to update your Funding Statement. Currently, your Funding Statement reads as follows:

“This study was supported by Provincial Program on Platform and Talent Development of the Department of Science and Technology of Guizhou China under Grant [No. [2019]5655 and [2019]5617], and the Guizhou Provincial Science and Technology Foundation under Grant (No. 2020–1Y096).”

Please include your amended statements within your cover letter; we will change the online submission form on your behalf.

Response: Thank you for your comment. We have removed any funding-related text from the manuscript and added the funding statements in cover letter.

4. Please include captions for your Supporting Information files at the end of your manuscript, and update any in-text citations to match accordingly. Please see our Supporting Information guidelines for more information: http://journals.plos.org/plosone/s/supporting-information.

Response: Thank you for your comment. We have included captions for Supporting Information files at the end of our manuscript, and updated any in-text citations to match accordingly.

5. We noticed you have some minor occurrence of overlapping text with the following previous publication, which needs to be addressed:

- https://journals.plos.org/plosone/article?id=10.1371/journal.pone.0239823

In your revision ensure you cite all your sources (including your own works), and quote or rephrase any duplicated text outside the methods section. Further consideration is dependent on these concerns being addressed

Response: Thank you for your comment. We have rephrased any duplicated text outside the methods section.

Reviewer(s)' Comments to Author:

Reviewer: 1

General comments

----------------

1. Phylogenetic analyses. In your phylogenetic analyses you should state more information about how the genes were chosen, how multiple sequence alignment was done, and the model of sequence evolution was chosen and applied. Some of this information is in the results (and should be moved to the methods). Lines 194-199 should be in the methods because they state which species were used and redundantly state how the tree was made. In the results you should state how many genes you used and detail information on the sequence matrices (informative sites, invariant sites, matrix size, etc.). When discussing relationships, you should state that taxa are "sister" to one another when they are each other’s closest relatives, not that they cluster together or are related. All species are related. I also point you towards a newer chloroplast phylogeney of the Dipsacales that includes multiple members of Lonicera:

Lee, A. K. et al. Reconstructing Dipsacales phylogeny using Angiosperms353: issues and insights. Am J Bot 108, 1122–1142 (2021).

Their paper assembles more Caprifoliacea cp genomes. In their analyses Weigela is the most distantly related of the taxa you survey for your phylogeny and should be used for rooting purposes. You should include information on branch length in your phylogeny, or state that it is a cladogram only to show the relationships and branch lengths we not infered.

Response: Thank you for your important comment. We have changed phylogenetic analyses and relative result parts in the revise manuscript. The detail changes as follow:

Phylogenetic analyses:

Phylogenetic analyses were performed between *L*. *ruprechtiana* and the other 22 cp genome sequences of the Caprifoliaceae (14 *Lonicera* species, 3 *Patrinia* species, 2 *Dipelta* species, 1 *Triosteum* species, 1 *Weigela* species, and 1 *Heptacodium* species) (S1 Table). All complete cp genomes were downloaded from NCBI (<https://www.ncbi.nlm.nih.gov/>). Only the homologous CDs (coding gene sequences) were used to construct phylogenetic tree to reduce data redundancy. A total of 68 homologous CDs, including *psbB*, *psbA*, *ndhB*, *rps7*, *rps12*, *psbA, matK, rps16, psbK, psbI, atpA, atpF, atpH, atpI, rps2, rpoC2, rpoC1, rpoB, petN, psbM, psbD, psbC, psbZ, rps14, ycf3, rps4, ndhJ, ndhC, atpE, atpB, rbcL, psaL, ycf4, cemA, petA, psbJ, psbF, psbE, petL, petG, psaJ, rpl33, rpl20, psbB, psbT, psbN, psbH, petB, petD, rps11, rpl36, infA, rps8, rpl14, rpl16, rpl22, rps19, rpl2, ndhF, rpl32, ccsA, ndhD, psaC, ndhE, ndhG, ndhI, ndhA,* and *ndhH*, were used to determine the phylogenetic relationship. Phylogenetic trees were constructed using the maximum-likelihood (ML) method (Model: Jones-Taylor-Thornton) with 1000 bootstrap replicates using MEGA7 [24].

Result part:

Phylogenetic analyses and whole cp genome sequence comparisons

To further understand the phylogenetic relationships of *L*. *ruprechtiana*, 68 homologous protein-coding genes of 22 cp genome sequences of the Caprifoliaceae were downloaded from NCBI database to build the phylogenetic tree. The phylogenetic tree was created using MEGA7 [24] with 1,000 bootstrap replicates. To show the relationships between *L*. *ruprechtiana* and other 22 family members, the phylogenetic tree is a cladogram and branch lengths we not infered (Fig. 3). As shown in Fig. 3, the cladogram clearly classified these 23 species into 2 distinct clusters. In the *Lonicera* cluster, *L*. *ruprechtiana* was fully resolved in a clade with *L. insularis* with a bootstrap support of 100% (Fig. 3), sister to three other species of *L. ferdinandi*, *L. vesicaria* and *L. maackii* (Fig. 3).

# We also changed the figure legend of figure 3:

# Figure 3. Cladogram summarizing the evolutionary relationships of 23 Caprifoliaceae species based on 68 homologous protein-coding genes of the chloroplast genomes. The maximum-likelihood tree shows the two distinct clusters. GenBank accession numbers are given in the figure. Shown next to the nodes are bootstrap support values based on 1,000 replicates.

We also cited this literature “Lee, A. K. et al. Reconstructing Dipsacales phylogeny using Angiosperms353: issues and insights” in the manuscript. The detail changes as follow:

The phylogenomic evolution of many cp genomes in Caprifoliaceae has recently been reported [17, 18]. 18: Lee, A. K. et al. Reconstructing Dipsacales phylogeny using Angiosperms353: issues and insights. Am J Bot 108, 1122–1142 (2021).

2. Comparison of L. japonica and L. ruprechtiana (L52-59). I do not know about the phytochemistry of these species but there are a few odd things in this paragraph. First I do not know what it means for L. ruprechtiana to be "a homologue to L. japonica" (L54). Homology has a clear definition in evolutionary biology and this sentence should be removed. There are multiple claims about the phytochemistry of L. ruprechtiana in comparison to L. japonica in this paragraph but none of them are cited. What is the evidence for these claims? Finally, the last sentence about introgression is not tied to the rest of the paragraph. It is not clear that a cp phylogeny alone will tell you about introgression, nor have you established a hypothesis of introgression between these species.

Response: Thank you for your important comment. We have rewritten this paragraph in the revise manuscript. We removed " *L. ruprechtiana* to be a homologue to *L. japonica* “and the last sentence of this paragraph. We also cited the phytochemistry of *L. ruprechtiana* in comparison to *L. japonica*. The detail changes as follow:

*Lonicera ruprechtiana* Regel is widely distributed in the east of the three provinces of Northeast China. Because of its excellent cold and drought resistance, it is often used as a greening tree species in northern China. *L*. *ruprechtiana* exhibit antibacterial effects that are no weaker than those of *L*. *japonica* and in some aspects superior [7]. Moreover, the biological activities and therapeutic effects of *L*. *ruprechtiana* are similar to those of *L*. *japonica* making it a possible substitute for *L*. *japonica* [7].

7: Zhu D, Zhao X, Dai L, Ji S. Experimental study on the pharmacology of *Lonicera ruprechtiana* Regel. Theory and Practice of Chinese Medicine. 2003; 2003: 111-112. (in chinese)

3. You should state the species you are comparing in the sections "Whole cp genome sequence comparisons" and "Synonymous and nonsynonymous substitution rate calculations".

Response: Thank you for your important comment. We have changed in the revise manuscript. The detail changes as follow:

**Whole cp genome sequence comparisons of *L. ruprechtiana*, *L. ferdinandi*, *L. vesicaria*, *L. maackii*, and *L. insularis***

**Synonymous and nonsynonymous substitution rate calculations of *L. ruprechtiana*, *L. ferdinandi*, *L. vesicaria*, *L. maackii*, and *L. insularis***

**Phylogenetic analyses and whole cp genome sequence comparisons of *L. ruprechtiana*, *L. ferdinandi*, *L. vesicaria*, *L. maackii*, and *L. insularis***

**Ks and Ka substitution rate analyses** **between *L. ruprechtiana* and the four other closely related species (*L. ferdinandi*, *L. vesicaria*, *L. maackii*, and *L. insularis*)**

4. ycf2 as a pseudogene. I am not familiar with the criteria that determine a pseudogene, but in L180 you state that ycf2 is an "essential pseudogene" because it exists in many repeats in the cp genome. What makes this locus an "essential pseudogene" (I don't know what essential means in this context either) and not simply a repetitive element belonging to a larger class such as LTR, SINE, etc?

Response: Thank you for your important comment. We have removed “essential pseudogene” in the revise manuscript.

5. L208-209 you state that your research is consistent with other research, but it is not clear in what way. Do you mean that coding regions are more conserved than non-coding regions? That is our expectation of sequence evolution.

Response: Thank you for your important comment. We mean that coding regions are more conserved than non-coding regions. We have changed in the revise manuscript. The detail changes as follow:

Our results mean that coding regions are more conserved than non-coding regions (Fig. 4), are consistent with those of earlier research [28].

----------

Line edits

----------

Abstract

- L26-27: Remove "and mVISTA", and change "conserved" to "largely conserved".

Response: Thank you for your comment. We have changed in the revise manuscript.

- L29: "genes under purifying" to "gene are under purifying"

Response: Thank you for your comment. We have changed in the revise manuscript.

Introduction

- L38: "large" to "larger"

Response: Thank you for your comment. We have changed in the revise manuscript.

- L43: Remove "(L. japonica)"

Response: Thank you for your comment. We have changed in the revise manuscript.

- L45-46: Remove "including" and place species in parentheses with "L." instead of "Lonicera"

Response: Thank you for your comment. We have changed in the revise manuscript.

- L60-61: Restructure. "The chloroplast (cp) genome is derived from the maternal parent and tends to exhibit a more highly conserved genomic structure than the nuclear genome."

Response: Thank you for your comment. We have changed in the revise manuscript.

Materials and methods

- L135: "was compared to four cp genomes in the Lonicera genus." to "was compared to four other Lonicera cp genomes."

Response: Thank you for your comment. We have changed in the revise manuscript.

- L139: Remove "among these related species."

Response: Thank you for your comment. We have changed in the revise manuscript.

Results

- L186: "Among these, the leucine codons had" to "Among these, leucine had"

Response: Thank you for your comment. We have changed in the revise manuscript.

- L187: "of cysteine codons was" to "of cystine was"

Response: Thank you for your comment. We have changed in the revise manuscript.

- L207-208: Remove ", indicating that the L. ruprechtiana cp genome underwent evolutionary divergence."

Response: Thank you for your comment. We have changed in the revise manuscript.

- L210: "conserved cp genes are" to "conserved cp genes were"

Response: Thank you for your comment. We have changed in the revise manuscript.

- L211: "obviously" to "most"

Response: Thank you for your comment. We have changed in the revise manuscript.

- L215: "relatively conserved" to "relatively more highly"

Response: Thank you for your comment. We have changed in the revise manuscript.

- L218: "being in the" to "being the"

Response: Thank you for your comment. We have changed in the revise manuscript.

- L226-228: Ka/Ks is only calculated relative to another sequence, so you should rephrase this sentence to state that Ka/Ks were calculated between L. ruprechtiana and the four other species. What are "sequence consistent genes"? Are these genes that had no polymorphisms? This sould be restated.

Response: Thank you for your comment. We have changed in the revise manuscript.

- L237: Remove "pressures"

Response: Thank you for your comment. We have changed in the revise manuscript.

Discussion

- L241: "with four other related Caprifoliaceae species" to "with four other closely related Lonicera species"

Response: Thank you for your comment. We have changed in the revise manuscript.

- L246: Remove "This means that the cp genomes of Lonicera species are conserved in length."

Response: Thank you for your comment. We have changed in the revise manuscript.

- L250: "sizes in" to "sizes of"

Response: Thank you for your comment. We have changed in the revise manuscript.

- L251: "four related Lonicera" to "four closely related Lonicera"

Response: Thank you for your comment. We have changed in the revise manuscript.

- L252: Remove "However, the gene types of the Lonicera cp genome were different." What are gene types? This sentence is too vague.

Response: Thank you for your comment. We have changed in the revise manuscript.

- L272: "preference is" to "preference was"

Response: Thank you for your comment. We have changed in the revise manuscript.

Reviewer: 2

The manuscript is well-organized and reports a study that uses contemporary methods to thoroughly examine the newly sequenced plastome of Lonicera ruprechtiana in the context of other complete plastomes from Caprifoliaceae family. This is a generally valuable study, like many other complete plastome studies that are currently published.

My main concern about the paper is on the assembly stats that are not adequately provided in the results, what was the N50 and the steps involved. Did The authors obtained a single contig in the with metaspades? I also hadn’t seen the use of the kraker for filtering cp and mt reads. The author really should clarify all of this issue.

Response: Thank you for your important opinion. We have changed the assembly result part in the revise manuscript. The detail changes as follow:

The Illumina sequencing platform produced 3,059 Mb raw data. After identifies mitochondrial and chloroplast sequences in original data by Kraken2 software, 1,525,022 organelle reads were acquired. After filtered, 2,268 Mb clean reads with a Q20 value of 96.6% were obtained. The metaSPAdes software was used to assemble the Clean Illumina reads. According to the software assembly results, there were 548 non-redundant contigs with 1,060,153 bp in length. The N50 value was 6,924 bp. Further analysis of the assembly results based on the reference genome using BLASTn software, we got a single contig. Then, we used the BLAST+ (2.9.0) software to analyze the covariance between *L. ruprechtiana* and the corresponding reference genome (S1 Fig). The result showed *L. ruprechtiana* and *L. sachalinensis* have strong covariance relationship (S1 Fig). Finally, we successfully construct the complete cp genomes of *L*. *ruprechtiana*.

I also think that PCR should have been done for verifying the junctions followed by Sanger sequencing. This would have helped in justifying the assembly.

If PCR was not done, I recommend the authors the needs to include a coverage analysis of the assembly to ensure accurate assembly of the chloroplast genome. Please include this important analysis in the paper.

Response: Thank you for your important opinion. we have done a coverage analysis of the assembly to ensure accurate assembly of the chloroplast genome. We used the blast+(2.9.0) software to analyze the covariance between *L. ruprechtiana* and the corresponding reference genome. The graph of covariance results is shown as Fig S1.

**S1_Fig.** Covariance plots. Red is the result of the covariance in the same direction; blue is the result of the covariance in the opposite direction.

In addition, since non-coding region are also powerful for phylogenetic resolution, were these regions tested in this case? If yes, was the result consistent with the one presented in the publication?

Response: Thank you for your important opinion. We did not use non-coding regions for the evolutionary tree, and as you suggested we tried to select non-coding sequences for the evolutionary tree, but since half of the genome is non-coding sequences, it was difficult to obtain sequences that occur in all 23 species.

The authors should also clarify the number of genes used to construct the phylogenetic tree.

Response: Thank you for your comment. We have changed in the revise manuscript.

The detail changes as follow:

Only the homologous CDs (coding gene sequences) were used to construct phylogenetic tree to reduce data redundancy. A total of 68 homologous CDs, including *psbB*, *psbA*, *ndhB*, *rps7*, *rps12*, *psbA, matK, rps16, psbK, psbI, atpA, atpF, atpH, atpI, rps2, rpoC2, rpoC1, rpoB, petN, psbM, psbD, psbC, psbZ, rps14, ycf3, rps4, ndhJ, ndhC, atpE, atpB, rbcL, psaL, ycf4, cemA, petA, psbJ, psbF, psbE, petL, petG, psaJ, rpl33, rpl20, psbB, psbT, psbN, psbH, petB, petD, rps11, rpl36, infA, rps8, rpl14, rpl16, rpl22, rps19, rpl2, ndhF, rpl32, ccsA, ndhD, psaC, ndhE, ndhG, ndhI, ndhA,* and *ndhH*, were used to determine the phylogenetic relationship.

The figures are also low quality and really should be improved.

Response: Thank you for your comment.

We have changed our figures to meet PLOS ONE's style requirements.
