# Supplementary material for: Characterization of the chloroplast genome of Lonicera ruprechtiana Regel and comparison with other selected species of Caprifoliaceae
Source: PLoS One. 2022 Jan 25;17(1):e0262813. doi: 10.1371/journal.pone.0262813 (PMC8789150; doi:10.1371/journal.pone.0262813)
Supplement: Attachment — Submitted filename: Response to Reviewers.docx [file pone.0262813.s008.docx]

December 31, 2021

Dr Branislav T. Šiler
Academic Editor 
PLOS ONE

Dear Editor and Reviewers,

RE: Response to Decision Letter for Manuscript ID. PONE-D-21-23416R1

First, thank you for your comments. We have now revised our manuscript further in accordance with your recommendations.

The following is the response to the comments of the academic editor and the reviewers with a detailed and clear point-by-point response.

We are looking forward to your positive decision.

Sincerely yours,

Qingbei Weng,

Ph.D, Professor

Department of Biotechnology

School of Life Sciences,

Guizhou Normal University, Guiyang, China

Manuscript ID. PONE-D-21-23416R1

Dear Dr. Weng,

Thank you for submitting your manuscript to PLOS ONE. After careful consideration, we feel that it has merit but does not fully meet PLOS ONE’s publication criteria as it currently stands. Therefore, we invite you to submit a revised version of the manuscript that addresses the points raised during the review process.

Additional authors' intervention regarding the phylogenetic analysis is required according to the Reviewer's recommendations. Moreover, the associated text should be considerably improved as it contains numerous inconsistencies. Generally, the whole manuscript should be thoroughly revised regarding proper language usage.

Please submit your revised manuscript by Jan 28 2022 11:59PM. If you will need more time than this to complete your revisions, please reply to this message or contact the journal office at plosone@plos.org. When you're ready to submit your revision, log on to https://www.editorialmanager.com/pone/ and select the 'Submissions Needing Revision' folder to locate your manuscript file.

Please include the following items when submitting your revised manuscript:

A rebuttal letter that responds to each point raised by the academic editor and reviewer(s). You should upload this letter as a separate file labeled 'Response to Reviewers'.

A marked-up copy of your manuscript that highlights changes made to the original version. You should upload this as a separate file labeled 'Revised Manuscript with Track Changes'.

An unmarked version of your revised paper without tracked changes. You should upload this as a separate file labeled 'Manuscript'.

If you would like to make changes to your financial disclosure, please include your updated statement in your cover letter. Guidelines for resubmitting your figure files are available below the reviewer comments at the end of this letter.

If applicable, we recommend that you deposit your laboratory protocols in protocols.io to enhance the reproducibility of your results. Protocols.io assigns your protocol its own identifier (DOI) so that it can be cited independently in the future. For instructions see: https://journals.plos.org/plosone/s/submission-guidelines#loc-laboratory-protocols. Additionally, PLOS ONE offers an option for publishing peer-reviewed Lab Protocol articles, which describe protocols hosted on protocols.io. Read more information on sharing protocols at https://plos.org/protocols?utm_medium=editorial-email&utm_source=authorletters&utm_campaign=protocols.

We look forward to receiving your revised manuscript.

Kind regards,

Branislav T. Šiler, Ph.D.

Academic Editor

PLOS ONE

Journal Requirements:

Please review your reference list to ensure that it is complete and correct. If you have cited papers that have been retracted, please include the rationale for doing so in the manuscript text, or remove these references and replace them with relevant current references. Any changes to the reference list should be mentioned in the rebuttal letter that accompanies your revised manuscript. If you need to cite a retracted article, indicate the article’s retracted status in the References list and also include a citation and full reference for the retraction notice.

Response: Thank you for your important comment. We have reviewed our reference list to ensure that it is complete and correct. We are not cited papers that have been retracted. There is one Chinese paper. Reviewer 1 had said there are multiple claims about the phytochemistry of *L. ruprechtiana* in comparison to *L. japonica* in this paragraph but none of them are cited. What is the evidence for these claims?” So, we add a Chinese paper:

7: Zhu D, Zhao X, Dai L, Ji S. Experimental study on the pharmacology of *Lonicera ruprechtiana* Regel. Theory and Practice of Chinese Medicine. 2003; 2003: 111-112. (in chinese)

This article was published in Chinese magazine, so I cited with “in Chinese” at the end of this paper.

We changed another chinese paper:

7: Wang G, Zhou X, Cui J, Zhao X, Yang X. Iridoid compounds in the buds of *Lonicera ruprechtiana* Regel. Chinese Journal of Medicinal Chemistry. 2009; 19: 206-208. (in chinese) (Web address: https://kns.cnki.net/kns8/defaultresult/index)

Reviewer(s)' Comments to Author:

Please use the space provided to explain your answers to the questions above. You may also include additional comments for the author, including concerns about dual publication, research ethics, or publication ethics. (Please upload your review as an attachment if it exceeds 20,000 characters)

Reviewer #1: Characterization of the chloroplast genome of Lonicera ruprechtiana Regel and comparison with other selected species of Caprifoliaceae

This paper presents the chloroplast genome of Lonicera ruprechtiana and conducts a few comparative genomic analyses (i.e., structure, selection, pairwise alignment) between L. ruprechtiana and other members of Lonicera and Caprifoliaceae, more broadly. The authors provide a valuable genomic resource for future studies of the Caprifoliaceae, particularly repetitive elements that may be used for population genetics. The authors have addressed most of the comments raised by reviewers, but the language is not particularly strong. I therefore have only minor comments about the analyses but provide numerous suggestions to improve the readability of the manuscript.

----------------

General comments

----------------

1. Phylogenetic analysis. There is still a major issue with the rooting of the phylogeny presented and the way the phylogeny is discussed. Weigela should be the root, as in all other phylogenetic analyses of Caprifoliaceae prior to this study. The members of the Caprifolieae (Lonicera, Triosteum, and Heptacodium) should form a clade sister to a clade containing Dipelta and Patrinia, with Weigela as the outgroup. The authors should comment on whether or not the phylogeny they present is consistent with previous work. Do the clades recovered make sense in comparison to the numerous plastid phylogenies for the Dipsacales have been published over the past 20 years?

Response: Thank you for your important comment. We have changed phylogenetic analyses using *Weigela* as root, and also changed relative result parts in the revise manuscript. The detail changes as follow:

“We used *Weigela* as the root, as in other phylogenetic analyses of Caprifoliaceae prior to this study[17, 18]. To show the relationships between *L*. *ruprechtiana* and other 22 family members, the phylogenetic tree is a cladogram and branch lengths we not infered (Fig 3). The members of the Caprifolieae (*Lonicera*, *Triosteum*, and *Heptacodium*) form a clade sister to a clade containing *Dipelta* and *Patrinia*, with *Weigela* as the outgroup (Fig 3). As shown in Fig. 3, *L*. *ruprechtiana* was sister to *L. insularis* with a bootstrap support of 100%.”

17.	Wang HX, Liu H, Moore MJ, Landrein S, Liu B, Zhu ZX, Wang HF. Plastid phylogenomic insights into the evolution of the Caprifoliaceae s.l. (Dipsacales). Mol Phylogenet Evol. 2020; 142: 106641.

18.	Lee AK, Gilman IS, Srivastav M, Lerner AD, Donoghue MJ, Clement WL. Reconstructing Dipsacales phylogeny using Angiosperms353: issues and insights. Am J Bot. 2021; 108: 1122-1142.

The language surrounding phylogenetics is still imprecise. Phylogenies are not generally used to classify clusters of species (although this does occur in pop gen and microbial studies), they represent hypotheses of the relatedness of the samples they contain. In lines 240-242, the author's incorrectly state that L. ruprechtiana is sister to 3 taxa. In fig 3 it is clear that L. ruprechtiana is sister to L. insularis. This needs to be revised. I would caution the authors from placing too much stock into this phylogeny because the sampling is very poor and this is almost certainly not a true sister relationship.

Response: Thank you for your comment. We have changed in the revise manuscript. The detail changes as follow:

“We used *Weigela* as the root, as in all other phylogenetic analyses of Caprifoliaceae prior to this study[17, 18]. To show the relationships between *L*. *ruprechtiana* and other 22 family members, the phylogenetic tree is a cladogram and branch lengths we not infered (Fig 3). The members of the Caprifolieae (*Lonicera*, *Triosteum*, and *Heptacodium*) form a clade sister to a clade containing *Dipelta* and *Patrinia*, with *Weigela* as the outgroup (Fig 3). As shown in Fig. 3, *L*. *ruprechtiana* was sister to *L. insularis* with a bootstrap support of 100%.”

I would also suggest moving the long list of genes from lines 132-137 into a table that shows missing data (i.e., which species you were able to gather which sequences from). In one of the comments to the reviewers the authors state that not all loci were recovered for all taxa, but we do not have any estimates of missing data.

Response: Thank you for your comment. We have added Table S2 to show which species were able to gather which sequences from.

“A total of 68 homologous CDs (S2 Table) were used to determine phylogenetic relationships.”

2. Selection analysis. More details need to be filled in the Methods and statistics need to be included in the results. What model was used? Did you exclude any codon positions? What window size did you use? Why did you choose the species that you did? They are very closely related and therefore have fewer mutations to consider. A comparison between species with more varying relationships might be more informative. Also, there are no test statistics included here. Because it is very difficult to recover 1 exactly (Ka = Ks), you will always classify genes as under one form or another of selection, when, in reality, the number is not distinguishable from 1. I would like to see test statistics associated with these estimates. The authors state that differences were found when comparing to different species for some genes, due to "evolutionary differences"; what are this difference? Are there reasons we might think these species/genes have been subject to different selection regimes?

More details need to be filled in the Methods and statistics need to be included in the results. What model was used? Did you exclude any codon positions? What window size did you use? Why did you choose the species that you did?

Response: Thank you for your comment. We have changed in the revise manuscript. The detail changes as follow:

Filled related parameters in the Methods:

“In addition, the mVISTA program (http://genome.lbl.gov/vista/mvista/submit.shtml) was used to compare to divergence across entire cp genomes with default settings (window size, 100bp; RepeatMasker, do not mask; RankVISTA probability threshold, 0.5).”

They are very closely related and therefore have fewer mutations to consider. A comparison between species with more varying relationships might be more informative.

Response: Thank you for your useful comment “A comparison between species with more varying relationships might be more informative”, In this work, we investigate divergence in the cp genome between *L*. *ruprechtiana* and the four other closely related species (*L*. *ferdinandi*, *L*. *vesicaria*, *L*. *maackii*, and *L*. *insularis*) because of these species had closely relationship. We should do the comparison between species with more varying relationships.

Also, there are no test statistics included here. Because it is very difficult to recover 1 exactly (Ka = Ks), you will always classify genes as under one form or another of selection, when, in reality, the number is not distinguishable from 1. I would like to see test statistics associated with these estimates.

Response: Thank you for your comment. We have added the test statistics in Table S5 (Synonymous (Ks) and non-synonymous (Ka) substitution rates of common coding genes between *L. ruprechtiana* and other four closed related species).

The authors state that differences were found when comparing to different species for some genes, due to "evolutionary differences"; what are this difference? Are there reasons we might think these species/genes have been subject to different selection regimes?

Response: Thank you for your comment. We have changed in the revise manuscript. The detail changes as follow:

“Some genes (*atpE*, *atpF*, *matK*, *ndhB*, *petB*, *petD*, *psaI*, and *rpl14*) had different Ka/Ks values (>1 or <1) under different comparisons (S5 Table), possibly due to these species/genes have been subject to different selection regimes.”

3. Pairwise alignment (Fig. S1). The results shown in Fig S1 should be refered to a pairwise alignment, not covariance, in the text. If I understand the pairwise alignment (synteny) analysis in Fig S1, the genomes mostly preserve synteny, however there is one large inverted repeat that should be noted in the results. Typically, in pairwise aligment plots like this, the red line should be broken where the blue lines are. What this plot seems to suggest is an inverted repeat. Is this correct? This should be discussed more in the results. See Fig. 1 of this paper on mammalian microinversions https://www.pnas.org/content/103/52/19824/tab-figures-data

Response: Thank you for your comment. We have changed in the revise manuscript. The detail changes as follow:

“Then, we used the BLAST+ (Version, 2.9.0) software to pairwise alignment between *L. ruprechtiana* and the corresponding reference genome (S1 Fig). As shown in S1 Fig, the genomes mostly preserve synteny, however there was one large inverted repeat (IRA and IRB regions, Fig 1); consistent with earlier research about mammalian evolution [28]”

28: Chaisson MJ, Raphael BJ, Pevzner PA. Microinversions in mammalian evolution. Proc Natl Acad Sci U S A. 2006; 103: 19824-9.

We also changed the figure legend of figure S1: “S1 Fig. Pairwise alignment plots. Red is the result in the same direction; blue is the result in the opposite direction”.

-------------------

Line edits/comments

-------------------

Abstract

- L17-18: "The chloroplast (cp) genome is a powerful tool for resolving genome evolution." This statement is tautological and should be removed.

Response: Thank you for your comment. We have changed in the revise manuscript.

- L27: "IR" needs to be defined before the abbreviation is used

Response: Thank you for your comment. We have changed in the revise manuscript.

- There is a mix throughout the abstract of the present and past tense. This should be resolved so that the results are written in the past tense.

Response: Thank you for your comment. We have changed in the revise manuscript.

The results in abstract are written in the past tense.

Introduction

- L39: "The Lonicera genus" to "Lonicera"

Response: Thank you for your comment. We have changed in the revise manuscript.

- L68-69: "Cp sequences have become a useful and powerful tool for revealing plant phylogenies [16]." is redundant with the two preceding sentences and should be removed.

Response: Thank you for your comment. We have changed in the revise manuscript.

We have removed this sentence.

- L70: Remove "phylogenomic"

Response: Thank you for your comment. We have changed in the revise manuscript.

- L77: "for the future genetic study" to "for future genetic studies"

Response: Thank you for your comment. We have changed in the revise manuscript.

Materials and Methods

- Throughout the M&M you do not need to write "software" after the name of the software

Response: Thank you for your comment. We have changed in the revise manuscript.

- L110: "of the related Lonicera" to "of Lonicera"

Response: Thank you for your comment. We have changed in the revise manuscript.

- L127-128: "between L. ruprechtiana and the other 22 cp genome sequences of the Caprifoliaceae" to "using the de novo L. ruprechtiana cp genome and 22 cp genomes from across the Caprifoliaceae"

Response: Thank you for your comment. We have changed in the revise manuscript.

- L137: "determine the phylogenetic relationship" to "determine phylogenetic relationships"

Response: Thank you for your comment. We have changed in the revise manuscript.

- L143: delete "in the Lonicera genus"

Response: Thank you for your comment. We have changed in the revise manuscript.

- L147: "divergence among the entire cp genome among these related species" to "divergence across entire cp genomes"

Response: Thank you for your comment. We have changed in the revise manuscript.

- I think it would be better to remove the species names from the subtitle "Synonymous and non-synonymous substitution rate calculations of L. ruprechtiana, L. ferdinandi, L. vesicaria, L. maackii, and L. insularis" and instead state the pairwise comparisons done in the body of the paragraph.

Response: Thank you for your comment. We have changed in the revise manuscript.

We have removed the species names.

- L153: "close" to "closely related"

Response: Thank you for your comment. We have changed in the revise manuscript.

Results

- It is not important to repeatedly mention that you used an Illumina platform, and I would suggest removing the repeated mentions of it

- As in the M&M, you should not write "software" after the name of each program

Response: Thank you for your comment. We have changed in the revise manuscript.

- L158: "identifies" to "identifying"

Response: Thank you for your comment. We have changed in the revise manuscript.

- L159: "in original data by" to "using"

Response: Thank you for your comment. We have changed in the revise manuscript.

- L160: "organelle" to "organellar" and "filtered" to "filtering"

Response: Thank you for your comment. We have changed in the revise manuscript.

- L162-162: "contigs with 1,060,153 bp in length. The N50 value was 6,924 bp." to "contigs totaling 1,060,153 bp in length and with an N50 of 6,924 bp."

Response: Thank you for your comment. We have changed in the revise manuscript.

- L164: What is the reference genome you mention here?

Response: Thank you for your comment. It means *Lonicera sachalinensis* (GenBank accession: MH028742), the reference sequences used for assembly the cp genome of *L. ruprechtiana*. We have changed in the revise manuscript. The detail changes as follow:“Further analysis of the assembly results based on the reference genome (*Lonicera sachalinensis*, the reference sequences used for assembly the cp genome of *L. ruprechtiana*) using BLASTn, we got a single contig.”

- L164-167: This section on comparative genomics should be moved to the subsection below on phylogenetics and comparative genomics. You should also state which program you used to the pairwise alignment between the two genomes; BLAST+ is the suite of all BLAST-related software, not an actual program iteself. Also see notes above on Fig. S1.

Response: Thank you for your comment. This section on comparative genomics state that how to assembly the cp genome of *L. ruprechtiana*, it may be more suitable in the result part of Cp genome assembly and genome features.

We have added the version number of Blast+ in the revise manuscript.

- L167-168: Delete "Finally, we successfully construct the complete cp genomes of L. ruprechtiana."

Response: Thank you for your comment. We have changed in the revise manuscript.

- L169: Are the IRA and IRB the repeats shown in Fig S1? If so, it should be referenced. You should also be referencing Fig. 1 when refering to these loci because it shows their positions and orientations.

Response: Thank you for your comment. IRA and IRB are the repeats shown in Fig S1. We have referenced in the revise manuscript.

- L177: "double" to "two"

Response: Thank you for your comment. We have changed in the revise manuscript.

- L190 (Table 1 caption): Delete "The detail"

Response: Thank you for your comment. We have changed in the revise manuscript.

- Table 1: "Construction of cp genome" to "Lengths of major regions"

Response: Thank you for your comment. We have changed in the revise manuscript.

- L208: "ranged" to "ranging"

Response: Thank you for your comment. We have changed in the revise manuscript.

- L209: "ranged" to "ranging"

Response: Thank you for your comment. We have changed in the revise manuscript.

- L213: "contributed by" to "contained in"

Response: Thank you for your comment. We have changed in the revise manuscript.

- L214: "intergenic region or partly in the gene spacer region" to "intergenic or spacer regions"

Response: Thank you for your comment. We have changed in the revise manuscript.

- L216 (Table 4 caption): Delete "Detail information of". I don't think Table 4 is important enough to take up so much room in the main text. I would suggest making it supplemental.

Response: Thank you for your comment. We have changed in the revise manuscript.

We have making Table 4 supplemental (S3 Table).

- L219: "sequences" to "sequences of the" and "produced" to "contained"

Response: Thank you for your comment. We have changed in the revise manuscript.

- L220-221: "Among these, the codon of leucine had the highest usage frequency, at 10.7%, while the usage frequency of cysteine was only 1.1% (S2 Table; Fig 2A)." to "Among these, leucine had the highest usage frequency, at 10.7%, while cysteine was least freuqent, at only 1.1% (S2 Table; Fig 2A)."

Response: Thank you for your comment. We have changed in the revise manuscript.

- L223: "with" to "had"

Response: Thank you for your comment. We have changed in the revise manuscript.

- L224: "in the L. ruprechtiana." to "by L. ruprechtiana"

Response: Thank you for your comment. We have changed in the revise manuscript.

- L225: "were" to "was"

Response: Thank you for your comment. We have changed in the revise manuscript.

- L227-230: The figure caption should be used to explain the figure, not tell results. I suggest simplifying the capture to "Percentage of amino acids of the L. ruprechtiana chloroplast (cp) genome (A) and the ending patterns of biased-usage codons (RSUC>1) (B)."

Response: Thank you for your comment. We have changed in the revise manuscript.

- L234-237: Rephrase "To further understand the phylogenetic relationships of L. ruprechtiana, 68 homologous protein-coding genes of 22 cp genome sequences of the Caprifoliaceae were downloaded from NCBI database to build the phylogenetic tree. The phylogenetic tree was created using MEGA7 [25] with 1,000 bootstrap replicates." to "To further understand the phylogenetic placement of L. ruprechtiana, 68 homologous protein-coding genes of 22 Caprifoliaceae cp genome sequences downloaded from NCBI were used to estimate a phylogeny using MEGA7 [25] with 1,000 bootstrap replicates (Fig. 3)."

Response: Thank you for your comment. We have changed in the revise manuscript.

- Delete lines 237-240 "To show... 2 distinct clusters." The statement that the authors only show a cladogram should be left for the figure caption, and see my notes on clusters above.

Response: Thank you for your comment. We have changed in the revise manuscript.

- L240: Delete "in the Lonicera cluster". Again, Lonicera a clade, not a cluster. The sentence also references Fig. 3 twice, and should only reference it once.

Response: Thank you for your comment. We have changed in the revise manuscript.

- L244-245: Delete "The maximum-likelihood tree shows the two distinct clusters."

Response: Thank you for your comment. We have changed in the revise manuscript.

- L246: Delete "in the figure"

Response: Thank you for your comment. We have changed in the revise manuscript.

- L250: "of five" to "of the five"

Response: Thank you for your comment. We have changed in the revise manuscript.

- L251: "the mVISTA program" to "mVISTA"

Response: Thank you for your comment. We have changed in the revise manuscript.

- L253: "mean" to "show" and "are" to "were"

Response: Thank you for your comment. We have changed in the revise manuscript.

- L254: "are consistent with those of" to "consistent with"

Response: Thank you for your comment. We have changed in the revise manuscript.

- L262: what does "were relatively more highly" mean? More highly what? It seems like something is missing here

Response: Thank you for your comment. We have changed in the revise manuscript.

The detail changes as follow: “were relatively more conservative than”

- L275-276: "were downloaded from GenBank and analyzed" to "are shown"

Response: Thank you for your comment. We have changed in the revise manuscript.

- L277: "negative-strand" to "reverse-strand"

Response: Thank you for your comment. We have changed in the revise manuscript.

- L283-284: "the evolution of genome and selection genes under pressure" to "selection pressure on genes and genomes"

Response: Thank you for your comment. We have changed in the revise manuscript.

- L294: "genes are strong" to "genes are under strong"

Response: Thank you for your comment. We have changed in the revise manuscript.

- L297: "the Illumina" to "an Illumina". There are many Illumina platforms

Response: Thank you for your comment. We have changed in the revise manuscript.

- L298: delete "others"

Response: Thank you for your comment. We have changed in the revise manuscript.

- L298-303 are meandering and repetitive. Possibly rephrase like "The complete cp genome of L. ruprechtiana showed a typical quadripartite cycle of 154,611 bp in length length, comparable to that of published Lonicera species cp genomes (154,513–155,346) (Fig. 1, Tables 1 and S1) [17]."

Response: Thank you for your comment. We have changed in the revise manuscript.

- L303: "variable" to "variation"

Response: Thank you for your comment. We have changed in the revise manuscript.

- 322: "the sequence of ycf2 gene have the highest variable" to "ycf2 is one of the most variable genes"

Response: Thank you for your comment. We have changed in the revise manuscript.

- L338: "the Illumina" to "an Illumina"

Response: Thank you for your comment. We have changed in the revise manuscript.

- L341: delete "information"

Response: Thank you for your comment. We have changed in the revise manuscript.

- L344: "had a close relationship with" to "is closely related to". See my note above; I caution over interpretation of this phylogeny

Response: Thank you for your comment. We have changed in the revise manuscript.

Supplement

- L469: "List of the cp genome of 23 species used for phylogenetic analysis." to "List of the 23 cp genomes used for phylogenetic analysis."

Response: Thank you for your comment. We have changed in the revise manuscript.

- L472: "rate" to "rates"

Response: Thank you for your comment. We have changed in the revise manuscript.

Reviewer #2: The authors have addressed all my concerns and therefore I support publication without further

changes.

Response: Thank you for your positive comment.
